# Exposure to trace amounts of sulfonylurea herbicide tribenuron-methyl causes male sterility in 17 species or subspecies of cruciferous plants

**DOI:** 10.1186/s12870-017-1019-1

**Published:** 2017-06-01

**Authors:** Cheng-Yu Yu, Jun-Gang Dong, Sheng-Wu Hu, Ai-Xia Xu

**Affiliations:** College of Agronomy, Northwest A&F University, 3 Taicheng Road, Yangling, Shaanxi 712100 China

**Keywords:** *Brassicaceae*, Male sterility, Gametocide, Sulfonylurea, Tribenuron-methyl

## Abstract

**Background:**

For most cruciferous plants, which are known as important crops and a number of weeds, hybrid breeding is hampered by the unavailability of a pollination control system. Male sterility induced by a gametocide can be useful for the utilization of plant heterosis.

**Results:**

The gametocidal effect of sulfonylurea herbicide tribenuron-methyl was tested across seventeen cruciferous species or subspecies including *Brassica juncea, B. carinata, B. oleracea* ssp. *capitata*, *B. oleracea* ssp. *acephala*, *B. rapa* ssp. *pekinensis, B. rapa* ssp. *chinensis, B. rapa* ssp. *parachinensis*, *B. nigra*, *Orychophragmus violaceus*, *Matthiola incana, Raphanus sativa*, *Sisymbrium altissimum*, *Eruca sativa*, *Sinapis alba, Sinapis arvensis, Capsella bursa-pastoris* and *Camelina sativa*. The plants of 23 cultivars in these species or subspecies were foliar sprayed with 10 ml of 0.2 or 0.4 mg/L of tribenuron-methyl before the vacuolated microspore formed in the largest flower buds; the application was repeated ten to twelve days afterwards. Tribenuron-methyl exposure significantly changed the flowering phenology and reproductive function. The treated plants demonstrated a one to four day delay in flowering time and a shortened duration of flowering, as well as other slight phytotoxic effects including a reduction in plant height and floral organ size. Approximately 80% to 100% male sterility, which was estimated by both pollen staining and selfing seed-set rate, was induced in the plants. As a result, plants were rendered functionally able to out-cross, with an average 87% and 54% manually pollinated seed-set rate compared to the corresponding controls at the 0.2 mg/L and 0.4 mg/L doses, respectively.

**Conclusions:**

The results suggested that male reproductive function was much more sensitive to tribenuron-methyl exposure than female function. This sulfonylurea herbicide has a promising use as the gametocide for hybrid production in cruciferous plants.

**Electronic supplementary material:**

The online version of this article (doi:10.1186/s12870-017-1019-1) contains supplementary material, which is available to authorized users.

## Background

Cruciferous plants (*Brassicaceae*) are known as important agricultural and horticultural crops, as well as a number of weeds, in both wild taxa and as escapees from cultivation. *Brassicaceae* species and varieties commonly used for food include cabbage *(Brassica oleracea)*, Chinese cabbage (*B. rapa* ssp. *pekinensis*), pak choi (*B. rapa* ssp. *chinensis*)*,* choy sum (*B. rapa* ssp. *parachinensis*), and radish (*Raphanus sativa*). Seeds of canola (*Brassica napus*), *Camelina sativa,* oriental mustard (*Brassica juncea*), Ethiopian mustard (*Brassica carinata*)*,* black mustard (*Brassica nigra*), yellow mustard (*Sinapis alba*), and rocket salad (*Eruca sativa*) are used in the production of canola oil and the condiment mustard. *B. oleracea* var. *acephala*, stock flower (*Matthiola incana*), and *Orychophragmus violaceus* are flowering and ornamental plants. In addition, due to the anticarcinogenic activity of glucosinolates and other bio-active compounds, *Brassicaceae* are involved in the pharmaceutical field [1]. Other species, including *Sinapis Arvensis*, *Sisymbrium Altissimum*, flixweed (*Descurainia Sophia*) and shepherd’s purse (*Capsella bursa-pastoris*), are prevalent weeds that compete with field crops, though *C. bursa-pastoris* is now domesticated as a vegetable in China.

Significant heterosis for biomass, seed yield and other agronomic traits have been well documented in some cultivated *Brassicaceae* species such as *Brassica* and radish, and hence, the use of hybrids is now an important technique for enhancing plant production and value [2–8]. The exploitation of male sterility (MS), achieved by chemical or genetic control, is the major approach to ensuring outcrossing in the female parent to economically produce F_1_ seed. Although genetically controlled MS systems that include cytoplasmic male sterility (CMS) and genic male sterility (GMS) are often effective and specific, for example, Ogura CMS and Polima CMS in *Brassica* [2], GMS in *B. rapa* [3, 4] and *B. oleracea* [5], and alloplasmic male sterilities invented in *B. juncea* [6–8], they require great pre-breeding efforts to create two or three different lines to induce, maintain or restore male sterility. Some MS-inducing cytoplasms also cause penalties in seed quality and other agronomically important traits such as organ morphology, growth habits, seed-set, branching patterns, or disease susceptibility [8]. For many other species, hybrid breeding is still limited by the lack of a practicable pollination control system such as CMS and GMS. The approach of chemically induced MS (CIMS), employing gametocides or chemical hybridizing agents to elicit MS in plants directly, requires little time- and labour-consuming pre-breeding work. Theoretically, almost any variety or breeding line could be induced to MS by an ideal gametocide and serve as the female parent to produce hybrid seed. An ideal gametocide should not cause significant environmental or health risks and should be economic and easy to apply. Most importantly, the treated plants should not be harmed by the gametocide. Thus, a highly effective and safe gametocide with low pollution is desirable for the utilization of heterosis.

An arsenate was used as a gametocide for rapeseed in China [9], but it was prohibited because it was considered an environmental pollutant. Detergent-induced MS was also reported in *B. juncea* [10] but not in other species. It was found that a foliar application of some sulfonylurea herbicides including tribenuron-methyl (trade name: Express) and amidosulfuron (Gratil) at very low doses resulted in over 95% MS of *B. napus* plants, as well as lower phytotoxicity on pistil fertility [11–14]. A method for hybrid seed production using cultivars susceptible to tribenuron-methyl as the female parent and using a resistant line as the male parent has been proposed [15, 16]. Up to now, 19 commercial rapeseed (*B. napus*) hybrids based on MS induced by tribenuron-methyl and other sulfonylurea herbicides have been registered in China. Indeed, CIMS is becoming an important approach to rapidly utilize the heterosis of the newly derived breeding lines in rapeseed. However, the gametocide has been seldom applied to other *Brassicaceae* species, except for those previously reported [17–19]. Tribenuron-methyl belongs to the sulfonylurea herbicide family of acetolactate synthase (ALS) inhibitors. ALS exists only in plants and some microbes but not in animals. ALS-inhibiting herbicides are widely used for weed control due to high crop selectivity, low application rates and low mammalian toxicity [20]. The present paper reports the results of a field investigation on the MS-inducing and phytotoxic effects of tribenuron-methyl on *Brassicaceae* species. Our results demonstrate that cruciferous plants exposed to trace amounts of tribenuron-methyl could show high rates of male sterility and out-crossing.

## Results

We investigated the influence of tribenuron-methyl exposure on six plant attributes including plant height (as the indicator of biomass), delay of flowering (DOF), duration of flowering (DUR), pollen viability (PV), self-pollinated seed-set (SSS), and manually pollinated seed-set (MPSS). Significant main effects of tribenuron-methyl dose and species (cultivars) and interactions between the main effects were observed (Table 1), indicating that the cultivars should be examined separately. Significant differences among the six biological attributes including plant height, DOF, DUR, PV, SSS, and MPSS under different doses were found (Table 1). The averages of the 6 attributes were significantly reduced by tribenuron-methyl exposure. A significant tribenuron-methyl × cultivar interaction existed due to obvious biomass differences among species and the possible inclusion of some species being insensitive to tribenuron-methyl. The six biological attributes from the three replicates of each cultivar were analysed for treatment effects by ANOVA (Additional file 1: Table S1). The detailed numerical raw data are shown in the subsequent figures. The tribenuron-methyl treatments produced significant changes in the plant attributes measured, though the effects varies greatly with species or cultivars (Additional file 1: Table S1). The results demonstrated that tribenuron-methyl could affect plant biomass (height) and fertility of all the studied native and weedy plant species.

**Table 1:** ANOVA results for plant response variables: height, delay of flowering (DOF), duration of flowering (DUR), pollen viability (PV), self-pollinated seed-set (SSS), and manually pollinated seed-set (MPSS)

Traits		Height		DOF		DUR		PV		SSS		MPSS	
	df	F	p	F	p	F	p	F	p	F	p	F	p
Replication	2	0.75	0.4743	0.06	0.9425	0.32	0.7246	0.11	0.8893	0.53	0.5906	0.01	0.9858
Dose	2	**76.48**	0.0001	**164.24**	0.0001	**14.34**	0.0001	**4832.65**	0.0001	**13.04**	0.0001	**114.48**	0.0001
Cultivar	22	**305.13**	0.0001	**3.61**	0.0001	**28.16**	0.0001	**1.85**	0.0410	1.01	0.4704	**66.96**	0.0001
Dose ×cultivar	44	**2.61**	0.0001	**3.88**	0.0001	**20.81**	0.0001	**3.36**	0.0001	**144.94**	0.0001	**6.57**	0.0001
Treatments		Average		Average		Average		Average		Average		Average	
Dose 0.0 mg/L		104.87	A	0.35	A	19.24	A	95.42	A	5.54	A	13.14	A
Dose 0.2 mg/L		101.32	B	1.30	B	18.62	B	5.91	B	0.13	B	11.91	B
Dose 0.4 mg/L		91.57	C	2.76	C	17.06	C	0.96	C	0.00	B	7.84	C

Values in bold indicate significance at *P* < 0.05. The differences among the average values of the 6 attributes for each treatment were tested by Duncan’s multiple range test and were indicated with letters A, B, and C

**Fig. 1:**
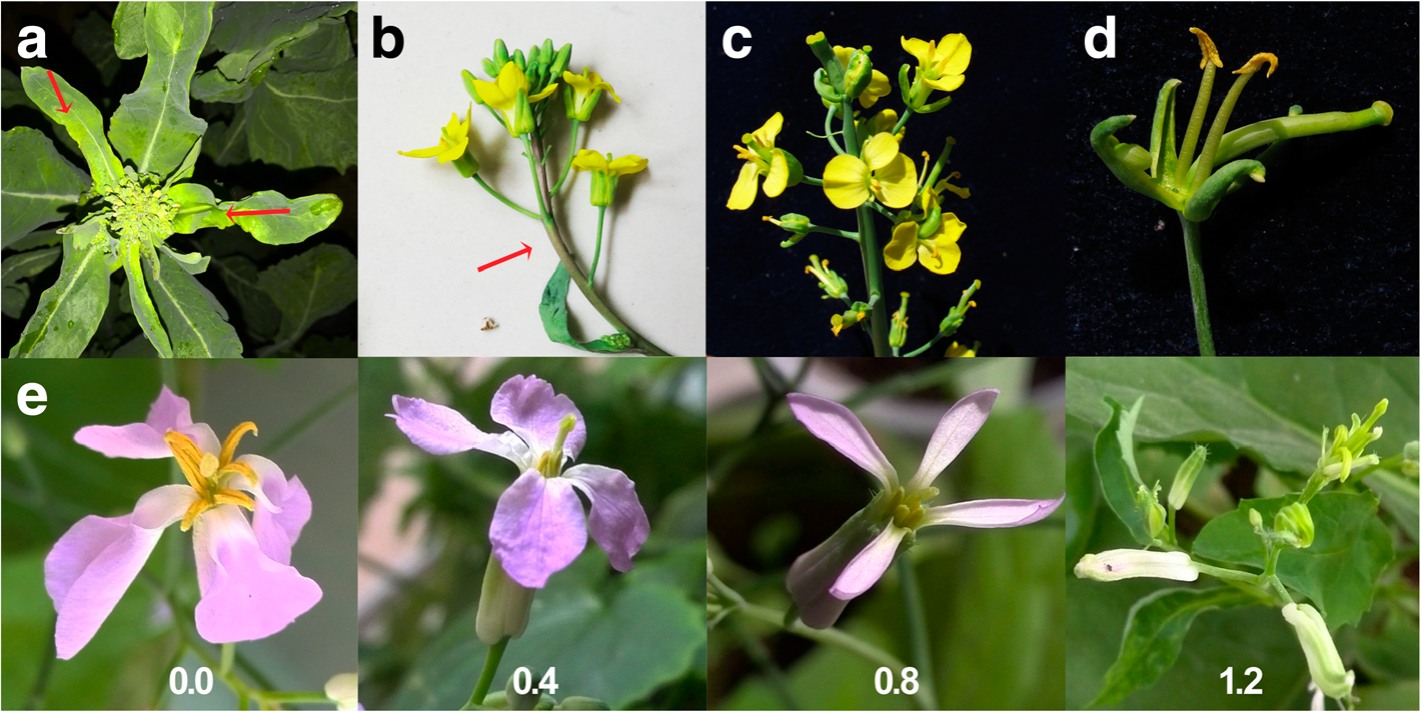
Damage on plants due to tribenuron-methyl exposure. **a** Chlorotic lesions at the site of contact within the first few days after treatment **b** Anthocyanidins accumulation in a young branch. **c** Malformed and male-sterile flowers after exposure to 1.2 mg/L of tribenuron-methyl. Note the lack of anthers and petals. **d** Magnified malformed and male-sterile flowers with the lack of anthers and petals. **e** Flowers of *Orychophragmus violaceus* treated with 0.0, 0.4, 0.8, and 1.2 mg/L of tribenuron-methyl*.* Higher doses had obvious toxicity to the size of the flower bud

**Fig. 2:**
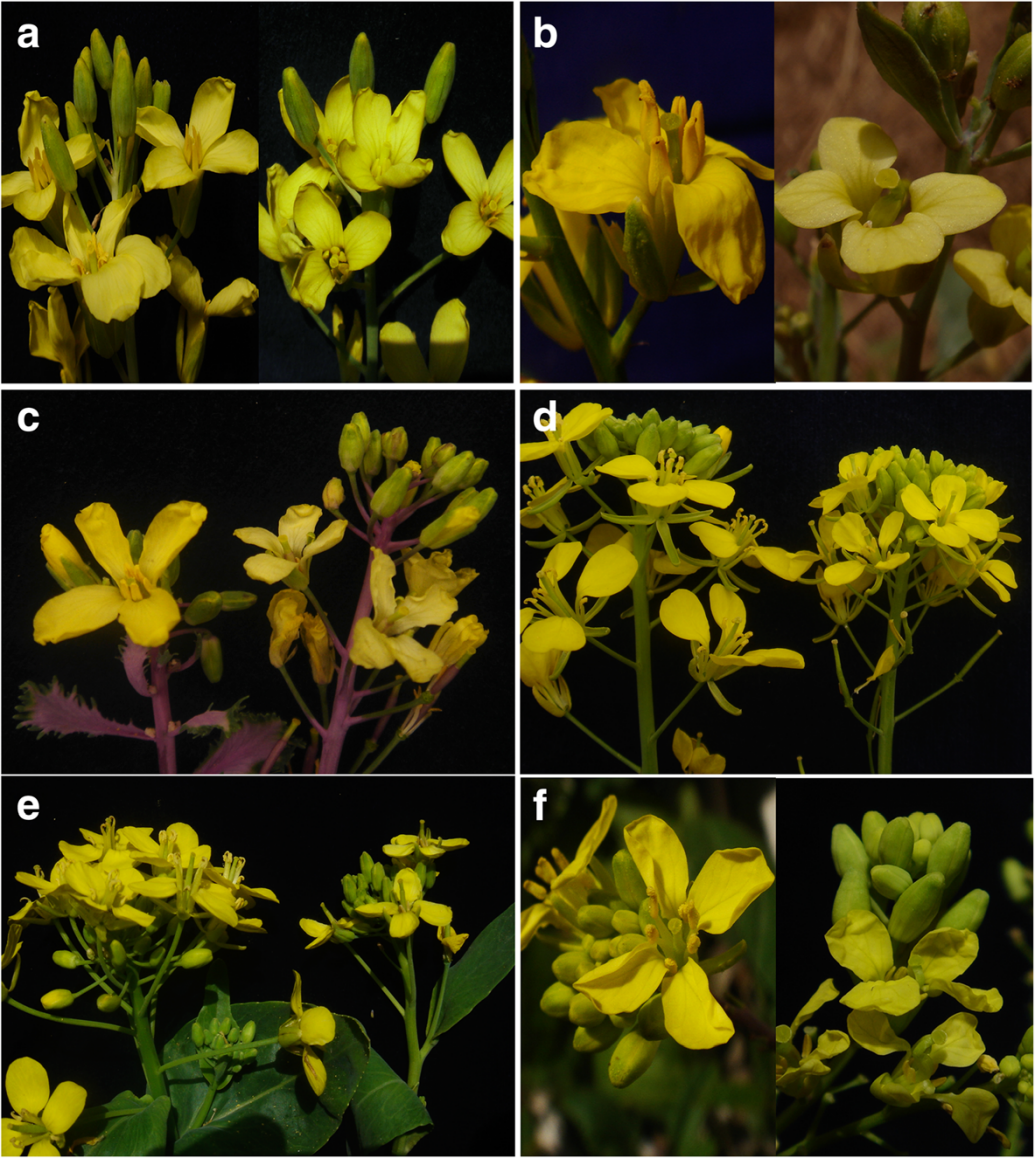
Male sterile flowers in *Brassica* species induced by 0.2 mg/L of tribenuron-methyl. The control is on the left side, and the tribenuron-methyl treatment is on the right side in each part. **a** *B. capitata*, **b** *B. carinata,* **c** *B. acephala*, **d** *B. juncea,* **e** *B. chinensis,* and **f** *B. nigra*

**Fig. 3:**
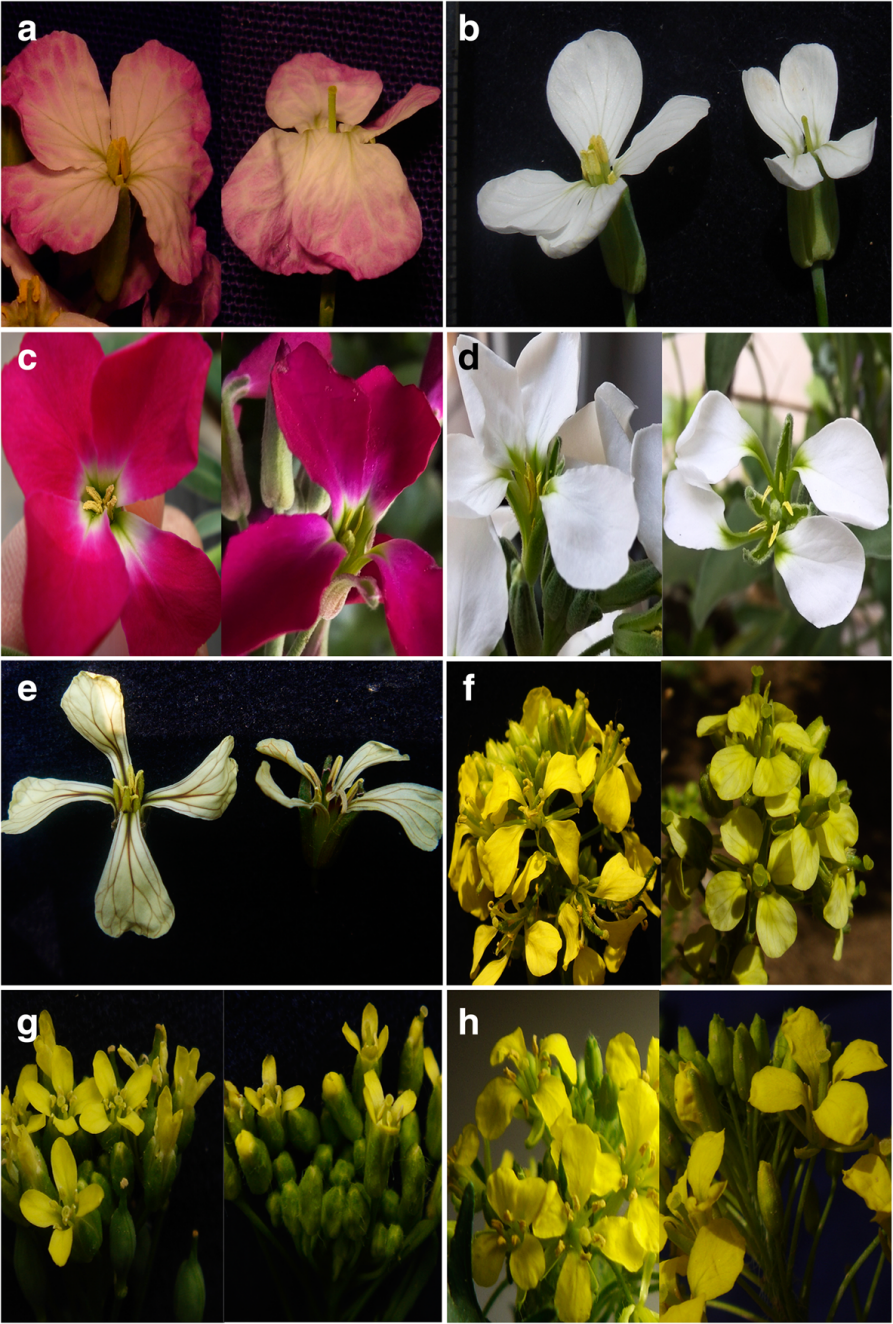
Male sterile flowers in the other crucifer species induced by 0.2 mg/L of tribenuron-methyl. The control is on the left side, and the tribenuron-methyl treatment is on the right side in each part. **a** Purple flower *R. sativa*, **b** White flower *R. sativa*, **c** Purple flower *M. incana,* **d** White flower *M. incana,* **e** *E. sativa*, **f** *S. alba,* **g** *C. sativa,* and **h** *S. altissimum*

Plants that were exposed to 0.2 or 0.4 mg/L of tribenuron-methyl developed slight damage on the vegetative tissues. The observed damage presented as chlorotic lesions on young leaves, and in some cases, the damage presented as the accumulation of anthocyanidins in young stems and siliques within the first few days after the treatment (Fig. 1a, b). The chlorotic lesions were evidence of a photosynthesis decline. After the initial plant damage, the 0.2 mg/L dose treated plants resumed vegetative growth without any further morphological indications of toxicity in the leaves and stem, except for the MS flower. Under the 0.4 mg/L treatment, the development of the meristems was suspended, but after a several day delay, the inflorescences resumed development. After exposure to 0.8 and 1.2 mg/L of tribenuron-methyl, plants demonstrated serious herbicide damage, which resulted in a reduction of plant height and biomass and deformed flowers that lacked petals and undeveloped stamens (Fig. 1c, d, and e). The 0.2 and 0.4 mg/L treatments typically resulted in thin and short anthers that appeared to be unable to properly dehisce and release pollen, and the filament was significantly shortened in all cultivars (Figs. 2 and 3). In contrast, the pistil morphology appeared to be more resistant to tribenuron-methyl damage because normal pistils were always present (Figs. 1, 2, and 3).

**Fig. 4:**
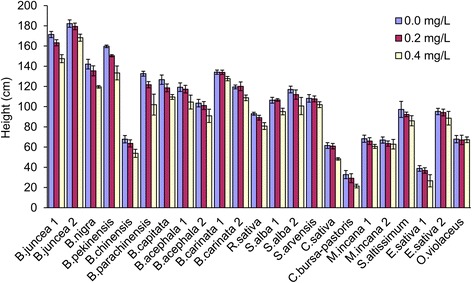
Plant height decreased by 0.2 and 0.4 mg/L tribenuron-methyl applications. Error bars represent ± one standard deviation

The treatment of 0.2 mg/L of tribenuron-methyl did not have a consistent effect on plant height. Plant height was not significantly reduced in *B. juncea*, *B. carinata, S. alba,* and *E. sativa*, though reductions were observed in most other species (Fig. 4). The treatment of 0.4 mg/L of tribenuron-methyl significantly reduced plant height in most species (*p* < 0.001). We assumed that the dose-species interaction was mainly caused by plant biomass differences because a larger and robust plant could be more tolerant to a higher pesticide dosage than a smaller one. However, the differences of the ALS gene and the pathway of metabolic detoxification could also be considered.

**Fig. 5:**
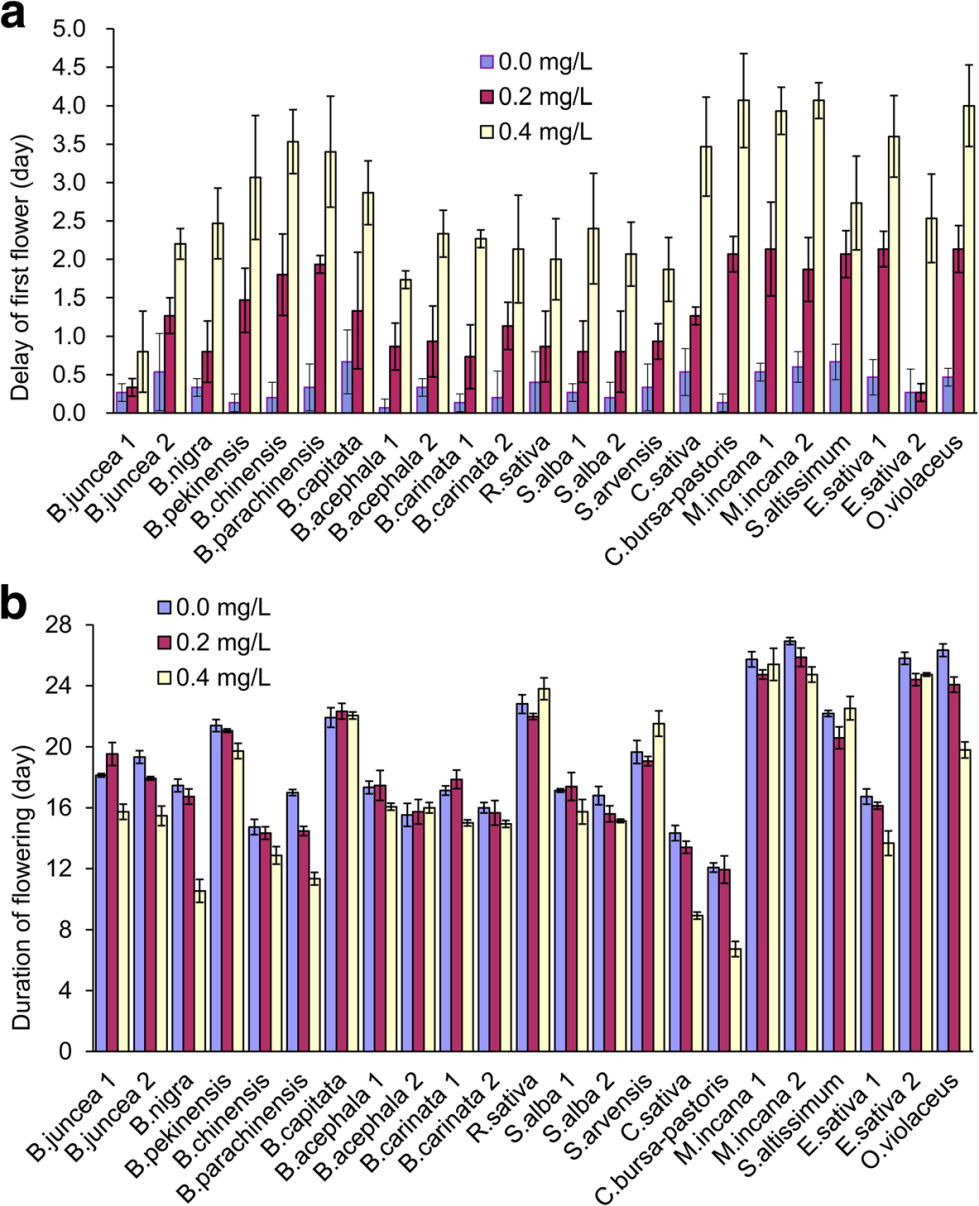
The delay of flowering (**a**) and the shortening of the duration of flowering (**b**) on different species resulting from 0.2 and 0.4 mg/L tribenuron-methyl applications. Error bars represent ± one standard deviation

The effects of tribenuron-methyl were assessed on the flowering phenology pattern by examining DOF (delay of flowering in a certain cultivar in comparison to the day when the first flower opened in any one of the nine plots growing the cultivar) and DUR (Fig. 5). The treatment significantly influenced both measurements, though not for all species or cultivars. The 0.2 mg/L treatment delayed the first flower by one day in *B. rapa, C. bursa-pastoris, M. incana,* and *S. altissimum* (Fig. 5), and the 0.4 mg/L treatment significantly delayed the first flower by two to four days in all cultivars. The 0.4 mg/L treatment also significantly reduced the duration of flowering for *B. nigra, B. rapa, C. sativa* and *C. bursa-pastoris*.

**Fig. 6:**
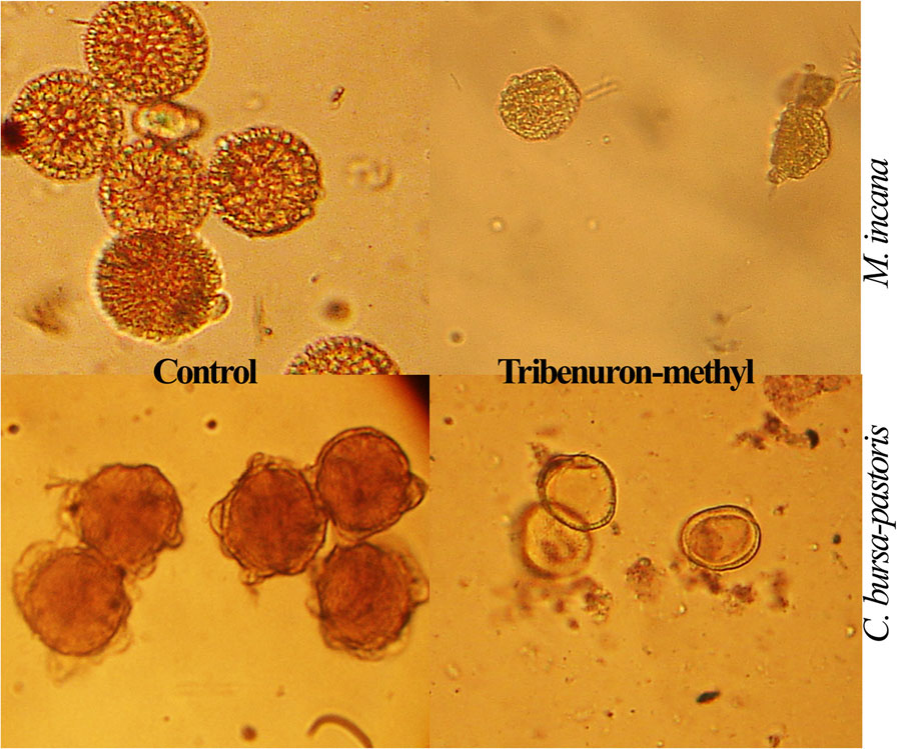
Pollen grains in *M. incana* and *C. bursa-pastoris* stained with acetocarmine. The pollen grains of the control are stained deeply; meanwhile, the aborted pollen grains cannot be stained, and the content disappeared

**Fig. 7:**
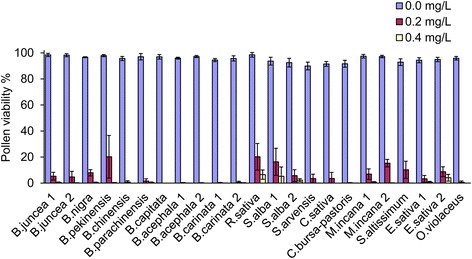
Effects of 0.2 and 0.4 mg/L tribenuron-methyl applications on the percentage of pollen viability. Error bars represent ± one standard deviation

To assess male sterility, the pollen viabilities of all treatments were estimated by using acetocarmine staining. Normal pollen grains stained deeply, with spherical shape and exine on the surface, while abortive pollen grains showed irregular shape and were unable to be stained due to a loss of cytoplasm and nuclear (Fig. 6). The proportion of stained pollen grains was significantly reduced by the tribenuron-methyl treatment. The average PV of the 0.2 mg/L treatment was 6% compared to the corresponding control, with a maximum of 20% in *B. pekinensis* and *R. sativa* (Fig. 7). The 0.2 mg/L treatment caused a satisfactory male sterile rate in most species, but it had an incomplete gametocidal effect on *B. pekinensis*, *R. sativa*, *S. alba* CV Veronica, *M. incana*, *S. altissimum*, and *E. sativa* CV Tokyo. The effect of the 0.4 mg/L treatment seemed much better in this species, since the average PV of the 0.4 mg/L treatment was reduced to 1% (Fig. 7).

**Fig. 8:**
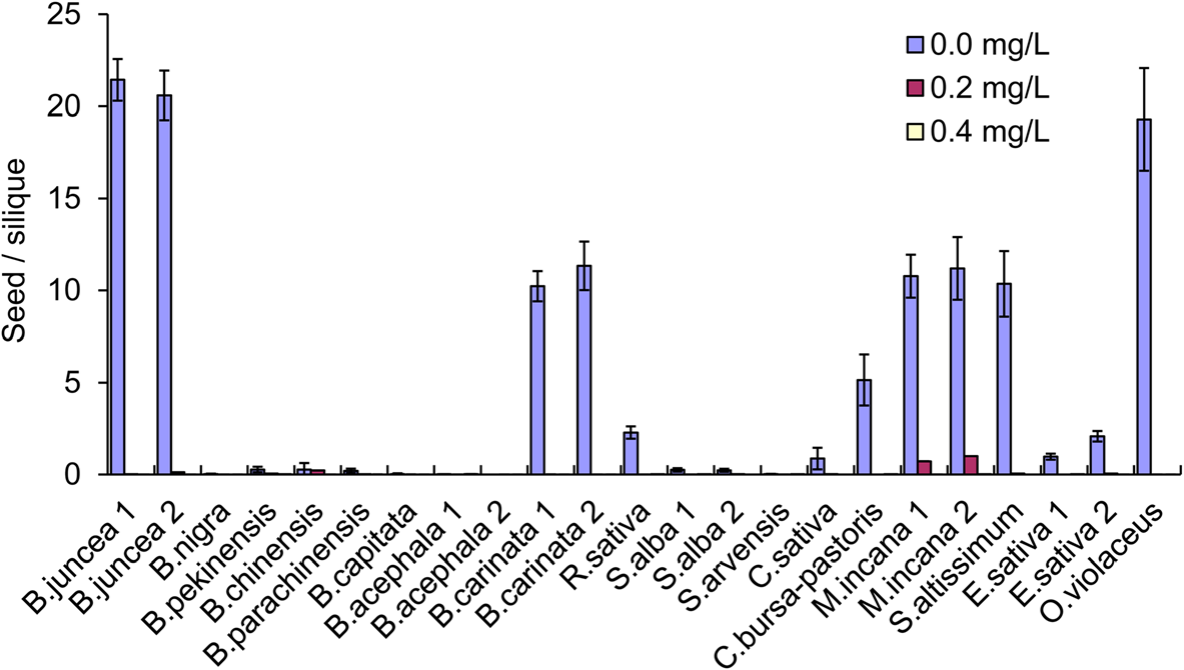
Effects of 0.2 and 0.4 mg/L tribenuron-methyl treatments on self-pollinated seed-set. Note: The lower rate of selfing seed-set in *B. nigra, B. oleracea, B. rapa,* and *S. arvensis* may be caused by both the treatments and self-incompatibility. Error bars represent ± one standard deviation

In addition to pollen viability, the ability of the plants to self-pollinate was examined in the 23 accessions. The proportion of successfully set seeds was significantly reduced by the tribenuron-methyl treatment. Except for *M. incana*, which formed an average of 1 seed/silique in the 0.2 mg/L treatment, the other treatment formed nearly no seed (Fig. 8) because there was not enough pollen for pollination in the male sterile plants. However, the decrease in selfing seed-set by self-incompatibility was also considered in species such as *B. nigra*, *B. oleracea, B. rapa,* and *S. arvensis*. Notwithstanding, the combined use of the gametocide and self-incompatibility in hybrid seed production fields may obtain purer hybrid seeds than the use of a single method.

**Fig. 9:**
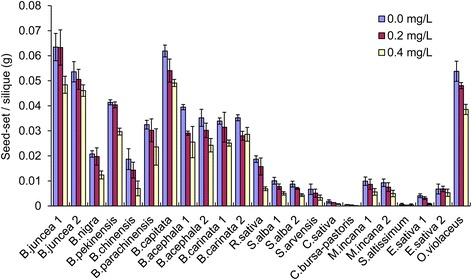
Effects of 0.2 and 0.4 mg/L tribenuron-methyl treatments on seed-set per silique under manual pollination. Error bars represent ± one standard deviation

Seed-set in plants under manual pollination was slightly reduced under the 0.2 mg/L treatment in most cases, with an average 87% seed-set rate compared to the corresponding control. The reduction was significant under the 0.4 mg/L treatment with an average seed-set rate of 54% for all cultivars but was 13%, 15%, 21%, and 33% for the puniest plants of *C. bursa-pastoris, E. sativa S. arvensis,* and *B. nigra* (Fig. 9). For treatments with a > 30% seed set rate under manual pollination, most obtained seeds were plump and could germinate to produce normal plants. However, approximately half of the seeds of the 0.4 mg/L treatment in *C. bursa-pastoris, E. sativa,* and *S. arvensis* that had poor seed set were wrinkled and malformed with lower seed viability (data not shown). In summary, the phytotoxic effects were not serious in some species with strong plant vigour and larger biomass. Relatively, high MS and low phytotoxicity were observed in *O. violaceus*, *B. juncea, B. carinata,* and *B. oleracea*, which often has an unusually stronger pistil and longer silique. Manual pollination could represent an initial success for hybrid seed production because we used other cultivars as the pollen donor in most tested species, except for *C. sativa and S. altissimum.*

## Discussion

### Prospect of an application of tribenuron-methyl as a gametocide in cruciferous plants

The results of our present study showed that the exposure of cruciferous plants to trace amounts of tribenuron-methyl caused developmental defects in the formation of stamens and other floral organs. Pollen viability was reduced to a very low level, but satisfactory female fertility was confirmed by manual cross-pollination. The results suggested that tribenuron-methyl could induce cruciferous plants to be highly sterile males while having comparatively low damage to female fitness. Therefore, the prospect of the application of tribenuron-methyl and other similar gametocides on cruciferous plant breeding seems to be promising. First, CIMS is a helpful complementary method for the utilization of heterosis, and the values are similar to those of the self-incompatibilities and environment-sensitive male sterilities. Second, in some ornamental plants and medicinal plants, where a heritable MS system is unavailable or where the introduction of foreign GMS or CMS will alter key biological attributes, CIMS could play an important role in hybrid breeding. Third, CIMS may make it easier to utilize hybrid vigour in minor specialty crops, which only needs a small scale for hybrid seed production with a carefully calibrated foliar application. Fourth, for forage grasses and green manure plants, which does not require high seed purity, a CIMS application for hybrid seed production would have little risk even if the hybridity of the obtained seed were not high. Fifth, CIMS could replace manual emasculation in conventional sexual hybridization and save time and labour cost for breeders or geneticists, who often make thousands of inter-variety crosses during the flowering stage to create recombined offspring or to evaluate hybrid performance. Lastly, distant hybridization could be promoted with the use of CIMS; for example, we successfully obtained dozens of hybrid seeds from a *B. carinata* MS plant induced by tribenuron-methyl and then pollinated by *B. napus* (unpublished).

*Brassicaceae* vegetables are highly regarded for their nutritional value. They provide high amounts of vitamin C and soluble fibre and contain nutrients with anticarcinogenic properties. For example, rocket salad or arugula are valuable nutrient resources; their spicy taste is widely used as a type of flavour in cooking, and they also have a long history of clinical use, as well as other types of use for various purposes [21]. Other plants, such as *B. carinata*, have several characteristics such as shatterproof siliques, tolerance to heat and drought, and resistance to blackleg disease [22]. Unfortunately, hybrid breeding for these crops is hampered by the unavailability of an MS system. A high level of MS induced by tribenuron-methyl brings an opportunity for the utilization of heterosis of these minor crops that belong to *Brassicaceae* including *S. alba, E. sativa, C. sativa, C. bursa-pastoris,* and *M. incana*. A previous study also reported that a foliar spray of 15 ng of tribenuron-methyl per plant of *A. thaliana* had a gametocidal effect [23]. In addition, exposure to trace concentrations of imazethapyr, an ALS-inhibitor, had unstable gametocidal effects on *Brassica* plants [13] and affected floral organ development and reproduction in *A. thaliana* [24]. Thus, an application of ALS-inhibiting gametocides on model plant *A. thaliana* to replace manual emasculation will be of interest to geneticists when they wish to make many genetic crosses. The results of the present study, combined with previous studies [12–14, 23, 24], suggest the possible use of other sulfonylurea chemicals as gametocides for cruciferous plants. The alternate use of other gametocides can resolve the concerns of insensitivity to tribenuron-methyl in some special genotypes. For example, amidosulfuron [12] had gametocidal effects similar to tribenuron-methyl in *B. napus* [11], *B. oleracea, B. juncea, B. carinata, B. juncea, O. violaceus,* and *S. alba* (data not shown).

### Considerations regarding gametocide application technology

Before the utilization of gametocide tribenuron-methyl [11], other gametocides were seldom applied in crucifer plants due to low efficiency or residual toxicity [9, 10]. Currently, a few experiments have been conducted to adjust the timing and dosage of gametocide applications [18, 19]. Li et al. [18] treated two cabbage self-incompatible lines MP01 and Y03 with 15 ml of a gametocide GS-1 solution, which contained a sulfonylurea herbicide and gibberellin, when the largest bud in the main raceme was approximately 2 mm long. They found that the concentration needed to ensure complete male sterility was 20 μg/mL for MP01 and 5 μg/mL GS-1 for Y03 [18]. In another study on *B. rapa*, a double spray of 3 to 6 ml of 10 mg/L of SX-1 (also contained tribenuron-methyl) solution at 10 to 12 day intervals resulted in over 95% male sterility [19]. It was also found that a double spray of 9 to 16 ml of 0.6 mg/L GSC (contained 15% tribenuron-methyl and 75% gibberellin) solution resulted in 98% male sterility [19]. Because gametocides including tribenuron-methyl showed no effects to nearly mature pollen, the first spray should be applied before microspore mitosis completion in the anther of the largest flower buds. Therefore, the right time for the first application of a gametocide is approximately 10–14 days before flowering Based on our experiences, the gametocidal effects of a single application of trace amounts of tribenuron-methyl could last for approximately two weeks (excluding the days from treatment to first flower opening) [11]. For species such as Shepherd’s purse (*C. bursa-pastoris*), which has a shorter duration of flowering (<15 days), less biomass, and earlier maturity, a single spray of tribenuron-methyl would be enough. However, for cruciferous such as cabbage, mustard, stock, and radish, which have large biomass, a long duration of blooming, and thick leaves covered by cuticle or trichomes that could impede the adhesion and uptake of a gametocide, a higher dose of tribenuron-methyl could be sprayed, but the risk of phytotoxic effects would rise. Alternatively, repeated applications at ten-day intervals could enhance and prolong the gametocidal effect.

Another technical problem is that the herbicide needs to be applied very evenly. Tribenuron-methyl and other sulfonylurea herbicides are rapidly absorbed by the foliage and roots and translocated throughout the plant. Thus, the proper dose to every treated plants needs to be carefully calibrated and distributed by a precise spray. A substitution for spraying is to smear or daub the gametocide on the stem of the plant [23] with a soft adhesive substance additive such as lanolin cream. Moreover, frequent rainfall at the bolting stage limits the application of a gametocide. The practicable measurements for the successful use of CIMS in hybrid breeding are as follows: growing the plants in a semi-arid area, the promotion of flowering, shortening the duration of flowering, and the addition of an adjuvant into the gametocide solution to accelerate foliar absorption. Therefore, the application techniques should be adjusted more or less when the gametocide is used on different female parents, in different areas and in different growing seasons.

### Concerns of gene flow changes in wild plants caused by an application of a sulfonylurea herbicide

Exposure to some herbicides such as glyphosate can alter a plant’s ability to produce seeds by affecting either male function, female function, or both [25]. In wild and weedy plants, changes in pollination patterns could contribute to changes in gene flow patterns between resistant and susceptible plants in that resistance alleles may move from resistant plants into male sterile susceptible plants [25]. Our results demonstrated that cruciferous plants exposed to sub-lethal doses of tribenuron-methyl could be subjected to very different pollination patterns (self-pollination vs cross-pollination). Thus, MS induced by sulfonylurea herbicides can affect gene flow in wild cruciferous plants. In fields that are being treated with sulfonylurea herbicides, specifically in feral conventional crops or sexually compatible weeds, weeds that are exposed to sub-lethal concentrations of herbicide are likely to be male-sterile and are therefore more likely to be cross-pollinated. This could have important implications for transgene confinement and management [25].

## Conclusion

Based on the obtained results, it can be concluded that a duplicate treatment with 0.2 and 0.4 mg/L of tribenuron-methyl appeared to be sufficient in inducing 80% to 100% male sterility in all tested species, especially *B. oleracea, B. carinata, B. juncea, B. rapa, B. nigra*, *O. violaceus*, *S. altissimum*, and *S. alba,* under the investigated conditions; there were advantages of higher efficacy, as well as low toxicity to pistil function at the application rates used. The results of the present study, combined with previous studies, suggest the possible use of tribenuron-methyl as the gametocide for hybrid breeding in cruciferous plants.

## Methods

### Plant materials and field experiment

A total of 23 germplasm collections belonging to 17 *Brassicaceae* species or subspecies (Additional file 1: Table S1) were tested here. In addition, we used other four cultivars including *B. nigra* CV GBRC39337, *S. arvensis* CV SAS*, C. bursa-pastoris* CV Shaoshaocai*,* and *R. sativa* CV Qingpi as the pollen donors for their counterparts in the same species. The original seeds of *B. carinata*, *B. nigra,* and *S. alba* were kindly provided by the Genebank of Crop Research Institute, Prague, Czech Republic (http://www.vurv.cz/). The original seeds of *R. sativa, B. acephala*, *B. pekinensis, B. chinensis, B. parachinensis. O. violaceus, M. incana* and *C. bursa-pastoris* were bought from Nongcheng Seeds Co., Ltd. (Yangling, China) and the seeds of *C. sativa* were provided by Kangfuduo Biotechnology Development Co., Ltd. (Beijing, China). The rest seed samples of *B. juncea, B. oleracea* ssp. *capitata*, *E. sativa, S. arvensis*, and *S. altissimum* were the breeding materials of the Northwest A&F University. A field trial was carried out in the breeding nursery of the Northwest A&F University, supported by the competent department of the government. The seeds of the most of the species with a winter growth habit were sowed in the isolated experimental field in autumn, and the plots were arranged in a randomized block design. Each treatment had three replications, and each plot contained approximately 20–35 plants. *R. sativa*, *B. oleracea* and *B. acephala* were sowed in August and then transplanted into the field in November. Four other species, *B. carinata, B. nigra, S. alba,* and *S. arvensis,* which are susceptible to freeze damage, were first cultivated in a greenhouse and then transplanted into the field in the early spring. More cruciferous plants were grown in the area surrounding the plots and used as pollen donors for the sterile plants. At the bolting stage (approximately 10 to 14 days before flowering and prior to the vacuolated microspore formed in the anther of the largest flower buds), each plant was sprayed with 10 ml solutions containing 0.0, 0.2, or 0.4 mg/L of tribenuron-methyl. We used manual sprayers to apply the solution evenly. The solution also contained 0.5% surfactant methyl oleate to ensure receiving and uptake of the correct amount of solution by the different plants. The treatments were repeated again ten to twelve days later. In some species, higher doses at 0.8 or 1.2 mg/L of tribenuron-methyl were also sprayed to observe the damage caused by an excessive application of the gametocide.

### Effects of the gametocide on agronomic and biological traits

Plant response variables including plant height (as the indicator of biomass), delay of flowering (DOF, the average time of first flower opening in the plot for a cultivar compared to the day when the first flower opened in any plant of the nine plots growing the cultivar), duration of flowering (DUR), pollen viability (PV), self-pollinated seed-set (SSS), and manually pollinated seed-set (MPSS) were recorded to estimate the phytotoxicity on growth and reproduction function.

The gametocidal effects of tribenuron-methyl on male function were evaluated by pollen viability, as well as by self-pollinated seed-set rate. The anthers in fresh flowers were collected from the stamens from at least five flowers per plant and five plants per treatment of all tested cultivars. The anthers were gently squeezed by forceps to release pollen grains into an aqueous solution with 1% acetocarmine dye, and the pollen viability (the proportion of stained pollen grains) was estimated under a light microscope [26]. In addition, we also evaluated anther function and pollen viability by measuring the possibility of seed-set under self-pollination, using at least five plants in each treatment that were isolated by paper bags and patted every day during the blossoming period. However, this method was not suitable for *B. nigra*, *B. oleracea, B. rapa,* and *S. arvensis* due to self-incompatibility.

The phytotoxic effects of tribenuron-methyl on female parts was estimated by measuring manually pollinated seed-set per silique. Twenty individual flowers were pollinated on at least five plants in each treatment, using mixed pollen grains collected from other plants of the same species. Manual pollination using pollen from sibling plants of the same cultivars was performed on *C. sativa* and *S. altissimum* due to only having one cultivar. *B. nigra, S. arvensis, C. bursa-pastoris* and *R. sativa* were manually pollinated by using pollen grains collected from cultivars *B. nigra* CV GBRC39337, *S. arvensis* CV SAS*, C. bursa-pastoris* CV Shaoshaocai*,* and *R. sativa* CV Qingpi, respectively, which were not included in this experiment due to an inadequate number of plants.

The data were analysed with ANOVA using Data Processing System software [27]. Our experimental factors included 3 doses, 23 cultivars, and 3 replicates. An examination of the simple treatment effects in each cultivar was also performed because the interaction effects among doses and cultivars were significant.

## Additional file

Additional file 1: Table S1. An ANOVA for the dose-response effects on the six plant attributes of each cultivar including height, delay of flowering (DOF), duration of flowering (DUR), pollen viability (PV), self-pollinated seed-set (SSS), and manually pollinated seed-set (MPSS). Values in bold indicate significance at *P* < 0.05 (DOC 60 kb)

## Abbreviations

ALS: Aacetolactate synthase
CIMS: Chemical-induced male sterility
CMS: Cytoplasmic male sterility
DOF: Delay of flowering
DUR: Duration of flowering
GMS: Genic male sterility
MPSS: Manually pollinated seed-set
MS: Male sterility
PV: Pollen viability
SSS: Self-pollinated seed-set
