# Supplementary material for: Exposure to trace amounts of sulfonylurea herbicide tribenuron-methyl causes male sterility in 17 species or subspecies of cruciferous plants
Source: BMC Plant Biol. 2017 Jun 1;17:95. doi: 10.1186/s12870-017-1019-1 (PMC5455082; doi:10.1186/s12870-017-1019-1)
Supplement: Additional file 1: Table S1. — An ANOVA for the dose-response effects on the six plant attributes of each cultivar including height, delay of flowering (DOF), duration of flowering (DUR), pollen viability (PV), self-pollinated seed-set (SSS), and manually pollinated seed-set (MPSS). Values in bold indicate significance at P < 0.05 (DOC 60 kb) [file 12870_2017_1019_MOESM1_ESM.doc]

**Table S1.** **ANOVA for dose-response effect on six plant attributes of each cultivar including height, delay of flowering (DOF), duration of flowering (DUR), pollen viability (PV), self-pollinated seed-set (SSS), and manual-pollinated seed-set (MPSS).** Values in bold indicate significance at P < 0.05.

| Species | Cultivars | Height | | DOF | | DUR | | PV | | SSS | | MPSS | |
| --- | --- | --- | --- | --- | --- | --- | --- | --- | --- | --- | --- | --- | --- |
|  |  | F | P | F | P | F | P | F | P | F | P | F | P |
| *B.juncea* | Wenxijie | **44.739** | 0.0018 | 2.375 | 0.2090 | **39.571** | 0.0023 | **2678.302** | 0.0001 | **1083.701** | 0.0001 | 5.778 | 0.0661 |
| *B.juncea* | Wugongjie | **13.480** | 0.0060 | **22.429** | 0.0067 | **44.603** | 0.0018 | **1104.960** | 0.0001 | **795.142** | 0.0001 | **14.409** | 0.0149 |
| *B.nigra* | RCAT0693 | **36.225** | 0.0027 | **28.300** | 0.0044 | **95.078** | 0.0004 | **4162.380** | 0.0001 | 1.000 | 0.4444 | **74.238** | 0.0007 |
| *B.pekinensis* | Qiubai | **26.602** | 0.0049 | **52.000** | 0.0014 | **15.217** | 0.0135 | **92.269** | 0.0005 | 0.641 | 0.5735 | **69.835** | 0.0008 |
| *B.chinensis* | Siyueman | **11.329** | 0.0225 | **75.040** | 0.0007 | **16.300** | 0.0119 | **13803.859** | 0.0001 | 1.896 | 0.2635 | **163.975** | 0.0001 |
| *B.parachinensis* | Caixin | **26.429** | 0.0049 | **24.813** | 0.0056 | **164.818** | 0.0001 | **2189.349** | 0.0001 | **7.300** | 0.0462 | **280.526** | 0.0001 |
| *B.capitata* | S25 | **15.078** | 0.0137 | **10.476** | 0.0257 | 0.5540 | 0.6130 | **9537.880** | 0.0001 | 1.000 | 0.4444 | **24.724** | 0.0056 |
| *B.acephala* | Huamudan | **42.333** | 0.0020 | **46.900** | 0.0017 | 3.331 | 0.1408 | **43257.540** | 0.0001 | 1.187 | 0.3937 | **8.235** | 0.0382 |
| *B.acephala* | Osaka | **26.4850** | 0.0049 | **22.571** | 0.0066 | 0.250 | 0.7901 | **53546.241** | 0.0001 | 1.000 | 0.4444 | **11.016** | 0.0236 |
| *B.carinata* | Sao Tome | **343.816** | 0.0001 | **51.062** | 0.0014 | **26.973** | 0.0048 | **18079.218** | 0.0001 | 1.187 | 0.3937 | **120.355** | 0.0003 |
| *B.carinata* | CQN4001 | **11.041** | 0.0235 | **11.473** | 0.0220 | 3.268 | 0.1441 | **4295.703** | 0.0001 | **220.869** | 0.0001 | **45.977** | 0.0017 |
| *R.sativa* | Guoguang | **43.324** | 0.0019 | **7.141** | 0.0479 | **8.714** | 0.0348 | **163.085** | 0.0001 | **142.552** | 0.0002 | 4.287 | 0.1012 |
| *S.alba* | Veronica | **29.225** | 0.0041 | **13.312** | 0.0171 | 3.230 | 0.1462 | **86.872** | 0.0005 | **19.043** | 0.0090 | **51.542** | 0.0014 |
| *S.alba* | Zlata | 5.992 | 0.0626 | **35.029** | 0.0029 | 6.836 | 0.0512 | **1198.290** | 0.0001 | **22.836** | 0.0065 | **39.470** | 0.0023 |
| *S.arvensis* | MB | **84.777** | 0.0005 | **11.352** | 0.0224 | **8.036** | 0.0397 | **1700.845** | 0.0001 | 1.187 | 0.3937 | **25.157** | 0.0054 |
| *C.sativa* | Jilan | **30.791** | 0.0037 | **48.400** | 0.0016 | **165.382** | 0.0001 | **1255.073** | 0.0001 | 6.224 | 0.0591 | **65.963** | 0.0009 |
| *C.bursa-pastoris* | Jicai | **16.127** | 0.0122 | **60.721** | 0.0010 | **102.361** | 0.0004 | **3346.911** | 0.0001 | **41.290** | 0.0021 | **48.952** | 0.0015 |
| *M.incana* | Purple ziluolan | **37.898** | 0.0025 | **39.455** | 0.0023 | 1.777 | 0.2804 | **1719.875** | 0.0001 | **244.031** | 0.0001 | **29.479** | 0.0040 |
| *M.incana* | White ziluolan | **10.408** | 0.0260 | **94.409** | 0.0004 | **11.671** | 0.0214 | **2129.0200** | 0.0001 | **137.568** | 0.0002 | **22.216** | 0.0068 |
| *S.altissimum* | Hornbach | 2.150 | 0.2322 | **16.326** | 0.0119 | **14.714** | 0.0143 | **373.5410** | 0.0001 | **99.658** | 0.0004 | **53.696** | 0.0013 |
| *E.sativa* | Choujie | **27.781** | 0.0045 | **50.273** | 0.0015 | **18.968** | 0.0091 | **1847.273** | 0.0001 | **109.342** | 0.0003 | **45.682** | 0.0018 |
| *E.sativa* | Tokyo | 1.728 | 0.2879 | **88.923** | 0.0005 | **22.563** | 0.0066 | **3252.659** | 0.0001 | **133.719** | 0.0002 | 5.476 | 0.0716 |
| *O.violaceus* | Eryuelan | 0.375 | 0.7090 | **63.909** | 0.0009 | **112.546** | 0.0003 | **7355.472** | 0.0001 | **144.046** | 0.0002 | **60.651** | 0.0010 |
